# Stimulation of GHRH Neuron Axon Growth by Leptin and Impact of Nutrition during Suckling in Mice

**DOI:** 10.3390/nu15051077

**Published:** 2023-02-21

**Authors:** Lyvianne Decourtye-Espiard, Maud Clemessy, Patricia Leneuve, Erik Mire, Tatiana Ledent, Yves Le Bouc, Laurent Kappeler

**Affiliations:** 1 INSERM, Centre de Recherche St-Antoine, Sorbonne Université, F-75012 Paris, France; 2 IHU-ICAN Institute of Cardiometabolism and Nutrition, F-75013 Paris, France

**Keywords:** linear growth, nutrition, somatotropic axis, leptin, hypothalamus, GHRH

## Abstract

Nutrition during the early postnatal period can program the growth trajectory and adult size. Nutritionally regulated hormones are strongly suspected to be involved in this physiological regulation. Linear growth during the postnatal period is regulated by the neuroendocrine somatotropic axis, whose development is first controlled by GHRH neurons of the hypothalamus. Leptin that is secreted by adipocytes in proportion to fat mass is one of the most widely studied nutritional factors, with a programming effect in the hypothalamus. However, it remains unclear whether leptin stimulates the development of GHRH neurons directly. Using a Ghrh-eGFP mouse model, we show here that leptin can directly stimulate the axonal growth of GHRH neurons in vitro in arcuate explant cultures. Moreover, GHRH neurons in arcuate explants harvested from underfed pups were insensitive to the induction of axonal growth by leptin, whereas AgRP neurons in these explants were responsive to leptin treatment. This insensitivity was associated with altered activating capacities of the three JAK2, AKT and ERK signaling pathways. These results suggest that leptin may be a direct effector of linear growth programming by nutrition, and that the GHRH neuronal subpopulation may display a specific response to leptin in cases of underfeeding.

## 1. Introduction

Numerous studies have highlighted the importance of nutrition during the early postnatal period for healthy development, in accordance with the developmental origin of health and adult diseases (DOHaD) hypothesis [1,2,3,4,5]. The development of the brain, and of the hypothalamus in particular, is particularly sensitive to changes in nutritional status, notably in the early postnatal period [6,7,8,9,10]. Physiological functions controlled by the hypothalamus can be altered following undernutrition during the suckling period, with effects on reproduction [11,12,13,14], food intake and metabolism [15,16,17,18], and linear growth [4,19].

Postnatal linear growth is regulated by the neuroendocrine somatotropic axis in mammals [4,20]. Within this axis, growth hormone (GH) is secreted by the somatotrophs of the pituitary gland. This secretion is tightly controlled by the growth hormone-releasing hormone (GHRH) and somatostatin (SRIH) neuropeptides, which are synthesized by the neurons of the arcuate (Arc) and periventricular (PeV) nuclei of the hypothalamus, respectively. SRIH inhibits GH secretion, whereas GHRH stimulates the secretion and synthesis of GH. Importantly, GHRH also stimulates somatotroph proliferation and differentiation during the early postnatal period, thereby programming the linear growth trajectory [21]. Indeed, undernutrition during the sucking period induces a permanent growth delay associated with a lowering of somatotropic axis activity [4]. In underfed pups, the innervation of the median eminence by GHRH neurons is delayed, leading to permanent somatotroph hypoplasia [4,19,22]. Developing GHRH neurons may, therefore, constitute a specific target of nutritional factors in the regulation of linear growth and establishment of adult size potential. Consistent with this hypothesis, the GH- and nutrition-dependent factor IGF-I has been shown to stimulate the axonal growth of GHRH neurons in a specific manner [19].

Leptin is secreted by adipocytes in proportion to the mass of white adipose tissues. It is the most widely studied of the nutritional factors potentially involved in hypothalamus development. Leptin has been reported to act on different neuronal populations, including the NPY neurons of the arcuate nucleus to control metabolism, and the ventral premammillary nucleus neurons to generate a permissive signal for reproduction [14,23,24]. However, the impact of leptin on the programming of linear growth remains unclear. Leptin signaling alterations have been associated with smaller adult size in humans [25,26]. Moreover, 45% of GHRH neurons express the leptin receptor, constituting a large population of Stat3+ neurons after leptin injection in rodent models [27]. In addition, mice with a knockout of the leptin receptor in cells expressing the GH receptor (GH-R)—including GHRH neurons as a first target for the negative feedback regulation of GH secretion—present a postnatal growth delay [28]. This suggests a possible direct role for leptin in the development of GHRH neurons, although no such role has been demonstrated to date. We therefore investigated whether leptin could directly regulate the axonal growth of GHRH neurons in the first few days of life.

## 2. Materials and Methods

### 2.1. Animal Experiments

All animal procedures were performed in accordance with institutional directives and EU directive 2010/63/EU for animal experiments and the care of laboratory animals. All procedures were approved by the French national ethics committee 05 Charles Darwin (Project protocol agreement number Ce5/2012/006 and APAFIS#8367-2016123008077830 vI). The mice in this study were housed under standard SOPF conditions, in individual ventilated cages at 22 °C under a 12 h light/12 h dark cycle, with free access to water and a standard chow diet for reproduction (LASQCdiet Rod18-R, sterilized; LASVendi, Soest, Germany) (Supplementary Table S1). All cages were enriched with commercial cotton nesting material. As previously described [19,29], C57Bl/6J Ghrh-eGFP male mice [30] were mated with wild-type C57Bl/6J females (Charles River Laboratories, L’Arbresle, France), and pregnant mice were housed individually to prevent any bias in pup nutrition during the suckling period, which extends from birth until the age of 16 days. At birth, pups were redistributed and cross-fostered such that there were five to six newborns per dam for normal milk feeding (normally fed), and nine newborns per dam in the group subjected to dietary restriction (underfed) [4,19,29,31]. All litters were submitted to the cross-fostering process. Pups were harvested from their original cage, individually rolled in the moistened dirty litter of the receiving dam, and then dried with a small piece of cotton from the nest of the receiving dam. Pups from the receiving dam were adopted by another one and eventual supernumerary newborns euthanized. Litters had globally equal numbers of male and female pups. Mice were studied at 7 and 10 days of age, depending on the experiment. Brains for explant cultures were harvested from seven-day-old pups after decapitation. Tissues and blood samples were harvested from 10-day-old mice under deep isoflurane anesthesia. The terminal blood sampling was performed by cardiac puncture with a 24G needle and a syringe coated with EDTA. Blood samples were centrifuged for 20 min at 2000 rpm at 4 °C. The plasma was isolated, frozen on dry ice and kept at −20 °C until use for individual biochemical determinations (see below). The number of mice or litters used is indicated in the results and figure legends. For all experiments and statistical analyses, the pups studied originated from at least three different litters, to prevent maternal bias.

### 2.2. Arcuate Explant Culture Experiments

Arcuate nuclei explants were prepared for culture in vitro, as previously described [19,29,32]. Briefly, brains were harvested from seven-day-old normally fed or underfed pups after rapid decapitation. For each experiment, brains from both male and female pups of the entire litter were pooled and processed together, to ensure that enough explant material was obtained for high-quality culture [19]. Thus, even though females are known to be less sensitive to nutritional restriction than males [4], which would decrease the magnitude of any effect observed, we harvested arcuate nucleus explants from pups of both sexes. We assessed eGFP expression in the median eminence directly, by inverted fluorescence microscopy (Evos Cell Imaging, Evos; Thermo Fisher, Wilmington, DE, USA). The brains were cut into 300 µm slices and incubated on membranes (Whatmann, Cytiva Europe GmbH, Velizy-Villacoublay, France) in MEM medium (Thermo Fisher Scientific) supplemented with 10% FCS, 1% glucose, and penicillin/streptomycin. Arcuate nuclei were then micro-dissected and cultured in neurobasal medium (Thermo Fisher Scientific) supplemented with methylcellulose, B-27 supplement (B-27) with insulin (#17504-044, Thermo Fisher Scientific), glucose, L-glutamine, and penicillin/streptomycin (Thermo Fisher Scientific). Cultures were performed in four-well plates, with each well filled with eight to nine explants. Arcuate explants from one litter of normally fed pups were plated on a single plate, whereas explants from one litter of underfed pups were plated in three plates. Explant experiments with normally fed and underfed pups were conducted sequentially, according to mouse production. The explants were cultured for 24 h, and were then left untreated (control condition) or were treated with 100 ng/mL leptin (6.25 nM, PeproTech, Neuilly-Sur-Seine, France) alone or in combination with 100 ng/mL IGF-1 (13.2 nM, R&D Systems, Abingdon, UK), 6.6 µM LY_294002 (LY, a PI3K inhibitor) (#1130, TOCRIS, Noyal Châtillon sur Seiche, France), 0.33 nM PD_0325901 (PD, a MAPK inhibitor) (#4192, TOCRIS), and/or 60 nM of NSC_33994 (NSC, a JAK2 inhibitor) (#4338, TOCRIS) for another 24 h. The culture was rinsed with PBS and fixed by incubation in 4% PFA for 30 min. The explants were then subjected to immunohistochemistry with primary antibodies directed against the leptin receptor, neurofilament (NF), eGFP/GHRH and/or AgRP, followed by the corresponding secondary antibodies (see Table 1 for antibody references and dilutions). All incubations were performed in 1x phosphate-buffered saline (PBS)/1% normal serum/0.05% tween 20. Neurofilament axons, eGFP/GHRH and AgRP axons were visualized under a 4X or 10X objective on a BX612 Olympus fluorescence microscope equipped with a DP71 using a charge-coupled device (CCD) camera or a BX43 Olympus equipped with a DP73 CCD camera, as indicated in figure legends. Leptin receptor labeling on Ghrh-eGFP+ axons was visualized under a 40x objective.

Axon length was analyzed with the NeuronJ plugin of ImageJ software, as previously described [19,29,33]. Briefly, we measured the lengths of up to 40 individual axons/explants and then calculated the mean length for each explant. The total axon length for each well was calculated from the mean for each explant. We accounted for variability of the explants cultures by normalizing axon growth in treated conditions against control conditions (untreated) on each plate, and presenting the results as a fold-difference. For statistical analyses, each plate was considered as a single experiment (*n* = 1). The number of plates studied in each experiment is indicated in the figure legends.

### 2.3. Biochemical Analysis

Plasma leptin concentrations in 10-day-old male mice were determined individually with the Mouse/Rat Leptin (R&D systems) ELISA kit, according to the manufacturer’s instructions. Absorbance was measured with a spectrophotometer (TECAN, GENios Pro).

For western-blot analysis, micro-dissected arcuate nuclei were harvested from one entire seven-day-old litter and processed in a separate set of experiments. The micro-dissected nuclei were incubated for 2 h in B-27-supplemented neurobasal medium and split into 2 pools, one of which was left untreated while the other was stimulated by incubation with leptin (100 ng/mL) for 15 min. Explants were rinsed with ice-cold PBS and total protein was extracted with NEB buffers in the presence of a cocktail of protease and phosphatase inhibitors (Roche), as previously described [19]. The explants from each litter were considered as a single sample (*n* = 1). For each sample, we separated 10 µg of cytoplasmic protein by electrophoresis in a NuPAGE Bis-Tris (4–12%) gel (Life Technologies), and the resulting bands were transferred to PVDF membranes. The membranes were then incubated with primary antibodies directed against total and serine-473-phosphorylated AKT, total and threonine-202/tyrosine-204-phosphorylated p44/42 MAPK (ERK1/2), total and serine-298-phosphorylated MAP2K1 (MEK1), total and tyrosine-1007/1008-phosphorylated JAK2, total and tyrosine-705-phosphorylated STAT3, and then with the corresponding secondary antibodies (see Table 2 for antibody references and dilutions). Antibody binding was detected with the Radiance Plus Femto Chemiluminescent Substrate (#AC2103, Azure Biosystems) or SuperSignal West Pico Chemiluminescent Substrate (#37070, Thermo Fisher Scientific), with a CCD camera on a ChemiDoc Touch Imaging System (Biorad). The membranes were then washed and incubated with an antibody against actin, which was detected with the SuperSignal West Pico Chemiluminescent Substrate, to allow for a normalization of loading between samples. The number of samples is indicated in the figure legends.

### 2.4. Statistical Analysis

All data are presented as the mean ± SEM. For explant cultures, the results of dual and triple immunohistochemistry (IHC) experiments were analyzed by two-way ANOVA with Bonferroni correction for multiple comparisons, and the results of single IHC were analyzed by one-way ANOVA with a Newman–Keuls multiple comparison test. Body weight, plasma leptin concentrations and Western blot results were analyzed in Mann–Whitney tests. Statistically significant results are marked with asterisks, * *p* < 0.05; ** *p* < 0.01; *** *p* < 0.001.

## 3. Results

**Figure 1:**
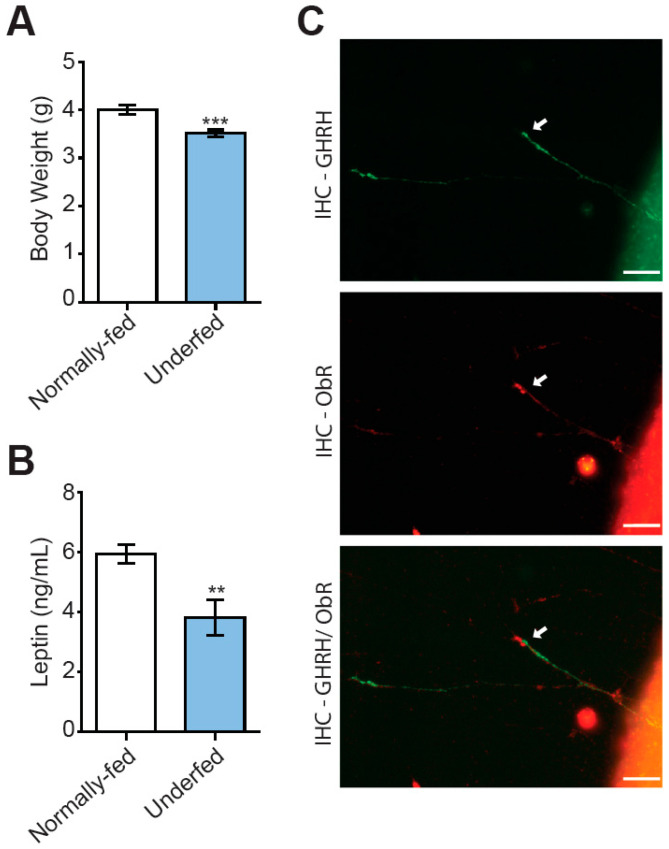
Underfeeding during suckling results in lower body weight and plasma leptin levels. (**A**) Increases in litter size from six (normally fed, open bars) to nine (underfed, blue bars) pups per dam are associated with a lower body weight in pups, by the age of seven days (*n* = 32 normally fed pups and 41 underfed pups of both sexes). (**B**) Underfeeding during suckling was associated with lower plasma levels of leptin at 10 days of age, as determined by ELISA (*n* = 8 per group). (**C**) Illustration (40X magnification obtained with a BX43 Olympus fluorescence microscope equipped with a DP73 CCD camera) of a growing axon from an arcuate nucleus explant cultured in vitro, showing that the GHRH+ axon in green (uppermost image) expresses the leptin receptor in red (middle image). A merged image is shown at the bottom (arrow). Scale bars represent 20 µm. Data are presented as the mean ± SEM. Comparisons were performed in Mann–Whitney tests, with ** *p* < 0.01 and *** *p* < 0.001.

The increase in litter size, from six to nine pups per dam, altered pup nutrition during the suckling period [4,19,29,31]. This resulted in delayed growth, which became evident in comparisons with age-matched normally fed pups at the age of seven days (Figure 1A) and was persistent throughout life, as previously reported [4,19]. Consistent with this finding, plasma leptin concentration was lower in 10-day-old underfed pups than in age-matched normally fed pups (Figure 1B).

We investigated the possibility that leptin might stimulate the development of GHRH neurons directly, by determining whether growing GHRH axons expressed the leptin receptor (Ob-R) in the Ghrh-eGFP mouse model [19,30], in which the identification of axons from GHRH neurons is simple. We confirmed, in our arcuate nuclei explants micro-dissected from seven-day-old normally fed Ghrh-eGFP pups and cultured in vitro, that growing axons of GHRH neurons expressed the leptin receptor (Figure 1C). Moreover, the distal part of growing GHRH+ axons and the growth cone of these axons appeared to be enriched in the leptin receptor, consistent with a potential role of leptin signaling in the axon growth of GHRH neurons.

### 3.1. Leptin Stimulates Axon Growth in GHRH Neurons in Arcuate Nucleus Explants from Normally Fed Pups

**Figure 2:**
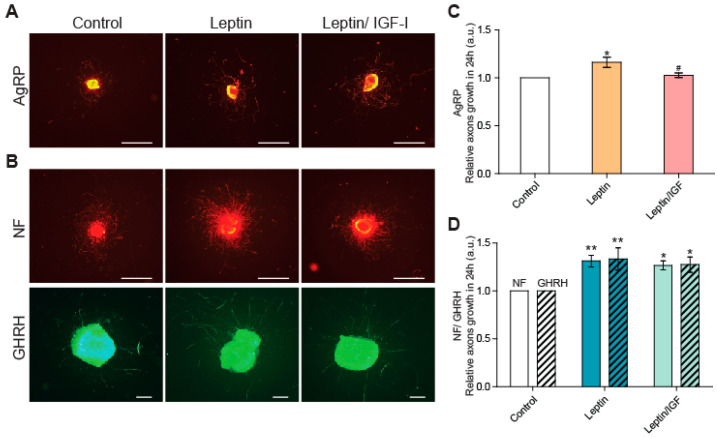
Leptin stimulates axon growth in GHRH neurons in arcuate nucleus explants from normally fed pups. (**A**) Illustrative IHC of AgRP neurons from arcuate nucleus explants micro-dissected from one-week-old normally fed pups, in control conditions (left panels), and after stimulation with leptin (middle panels) and with leptin/IGF-1 (right panels). (**B**) Illustrative images of dual IHC for the axons of total arcuate nucleus neurons labeled with NF (top panels in red) and GHRH neurons labeled with eGFP (bottom panels in green), in the same conditions. Scale bars represent 1000 µm for AgRP+ and NF+ IHC (4X magnification) and 200 µm for GHRH+ IHC (10X magnification), for images from a BX612 Olympus fluorescence microscope equipped with a DP71 CCD camera. (**C**) Quantification of the growth of AgRP axons after 24 h of stimulation with leptin or leptin/IGF-I relative to control conditions (*n* = 4 experiments), and (**D**) of the growth of NF (plain bars) and GHRH (dashed bars) axons (*n* = 5–7 experiments). Data are presented as the mean ± SEM. Results were compared in a one-way ANOVA with the Newman–Keuls post hoc test (c) or a two-way ANOVA with Bonferroni correction (d), with *: *p* < 0.05 and **: *p* < 0.01 vs. control conditions and #: *p* < 0.05 vs. leptin stimulation.

We then investigated whether leptin could regulate axon growth in arcuate neurons, using arcuate nucleus explants harvested from seven-day-old normally fed pups. Leptin was initially reported to stimulate axon growth in AgRP/NPY+ orexigenic neurons [15,34]. We therefore first confirmed that we were able to measure this stimulation in the 24 h treatment conditions applied in this study. The AgRP+ labeled axons treated with leptin for 24 h were 1.16 ± 0.06 times longer than the control (untreated) axons, with lengths of 541 ± 88 µm in control conditions and 627 ± 98 µm following leptin stimulation (*n* = 4 experiments, *p* < 0.05) (Figure 2A,C).

All the neurons of the arcuate nuclei were labeled with an antibody directed against the ubiquitously expressed neurofilament protein (NF), and a stimulatory effect of leptin was visible in the total neuron populations of arcuate nuclei (Figure 2B,D). Treatment with leptin for 24 h resulted in arcuate neuron axons that were 1.31 ± 0.07 times longer than those observed in control conditions: 484 ± 45 µm for control conditions and 628 ± 49 µm following leptin treatment (*n* = 7 experiments, *p* < 0.01). We then looked at the specific effects of leptin on axon growth in the subpopulation of GHRH neurons. In the explants used for the NF analysis described above, we found that Ghrh-eGFP+ axons treated with leptin were 1.33 ± 0.13 times longer than control axons: 385 ± 27 µm in control conditions and 510 ± 47 µm after leptin treatment (*n* = 5 experiments, *p* < 0.01) (Figure 2B,D). These results suggest that leptin levels may directly regulate the development of GHRH neurons. We previously showed that IGF-1 selectively stimulates axon growth in GHRH neurons [19]. We therefore investigated the possible synergy between leptin and IGF-1. The treatment of arcuate nucleus explants with both leptin and IGF-I for 24 h did not lead to any additional increase in axon length relative to leptin treatment alone, either in the total neuron population of the arcuate nucleus or in GHRH neurons (Figure 2C,D). Similar results were obtained for AgRP neurons.

**Figure 3:**
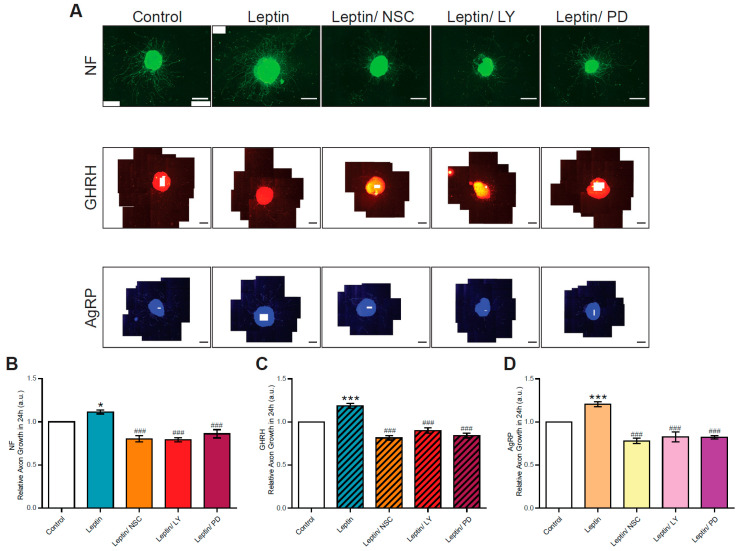
Signaling pathways involved in the axon growth of arcuate neurons in explants from normally fed pups. (**A**) Illustrative triple IHC of arcuate nucleus explants from the hypothalamus micro-dissected from one-week-old normally fed pups under control conditions (first panel), and following stimulation with leptin (second panel), leptin/NSC (third panel), leptin/LY (fourth panel) and leptin/PD (fifth panel), with NF (top panels in green), GHRH (middle panels in red) and AgRP (bottom panels in blue). Scale bars represent 100 µm for NF+ IHC (4X magnification) and 200 µm for GHRH+ and AgRP+ IHC (10X magnification), on images obtained with an Olympus BX43 fluorescence microscope equipped with a DP73 CCD camera. Quantifications of the growth of (**B**) NF (*n* = 5–9 experiments), (**C**) GHRH (*n* = 4–10 experiments) and (**D**) AgRP (*n* = 5–10 experiments) axons stimulated for 24 h with leptin alone, or in combination with one of the three inhibitors (NSC_33994, LY_294002 or PD_0325901). Data are presented as the mean ± SEM. Results were analyzed by two-way ANOVA with Bonferroni correction, with *: *p* < 0.05 and ***: *p* < 0.001 vs. control conditions and ###: *p* < 0.001 vs. leptin stimulation.

The absence of a synergic stimulatory effect of IGF-I and leptin on axon growth in GHRH neurons suggests that these two molecules may use the same signaling pathways. Indeed, IGF-I is known to act through its IGF-type 1 receptor (IGF-1R) and the AKT and MAPK signaling pathways. By contrast, leptin binds to its specific receptor (Ob-R), which is a class 1 cytokine receptor, and acts by stimulating the JAK-STAT signaling pathway. Connections between the JAK-STAT signaling pathway and the AKT and the MAPK signaling pathways have been reported [10,15,35]. We investigated whether a specific leptin-stimulated signaling pathway was involved in axonal growth by treating arcuate nucleus explant cultures with both leptin and a specific inhibitor of JAK-STAT (NSC), PI3K-AKT (LY) or MAPK (PD) signaling. The treatment of explants with leptin plus one of these inhibitors (NSC/LY/or PD) significantly decreased (*p* < 0.001) the leptin-induced axon growth of arcuate neurons relative to stimulation with leptin alone (Figure 3).

For the total (NF+) arcuate neuron population, axon growth decreased to 0.85 ± 0.04 (leptin/NSC), 0.79 ± 0.03 (leptin/LY) and 0.86 ± 0.05 (leptin/PD)-times shorter than that in control conditions (Figure 3A,B). Similarly, for GHRH neurons, the axon growth decreased to levels of 0.84 ± 0.03-, 0.85 ± 0.06- and 0.84 ± 0.03-fold those in control conditions, respectively (Figure 3A,C). Similar results were obtained with the AgRP+ neuron population: a decrease in axon growth to 0.80 ± 0.04-, 0.83 ± 0.06- and 0.79 ± 0.04-fold control levels, respectively (Figure 3A,D). Overall, these results suggest that the JAK/STAT, AKT and MAPK signaling pathways are all important for the leptin-induced growth of axons in arcuate nucleus neurons, including GHRH neurons.

### 3.2. GHRH Neurons in Arcuate Nucleus Explants from Underfed Pups Do Not Respond to Leptin Stimulation

**Figure 4:**
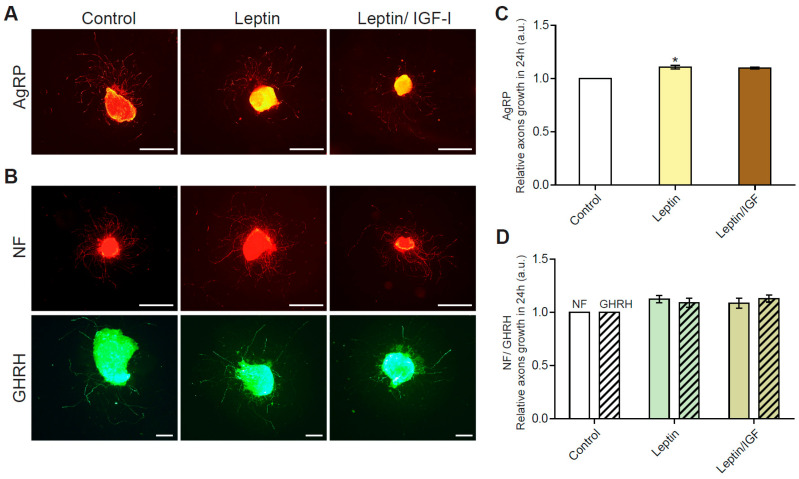
GHRH neurons from underfed pups are resistant to leptin for the stimulation of axon growth. (**A**) Illustrative IHC of arcuate nucleus explants from the hypothalamus micro-dissected from one-week-old underfed pups in control conditions (left panel), and after stimulation with leptin alone (middle panel), or with leptin/IGF-1 (right panel), with AgRP axons labeled in orange. (**B**) Axons from total arcuate nucleus neurons and GHRH neurons labeled by dual-IHC for neurofilament (NF, in red) and eGFP (in green), respectively. Scale bars represent 1000 µm for AgRP+ and NF+ IHC (4X magnification) and 200 µm for GHRH+ IHC (10X magnification), on images from an Olympus BX612 fluorescence microscope equipped with a DP71 CCD camera. (**C**) Quantification of the growth of AgRP axons stimulated by incubation for 24 h with leptin or leptin/IGF-I, relative to control conditions (*n* = 5 experiments), and (**D**) quantification of the growth of NF (plain bars) and GHRH (dashed bars) axons (*n* = 6 experiments). Data are presented as the mean ± SEM. Results were compared in a one-way ANOVA with the Newman–Keuls post hoc test (c) or in a two-way ANOVA with Bonferroni correction (d), with *: *p* < 0.05.

We previously reported that underfeeding during suckling was associated with a resistance of GHRH neurons to IGF-I-induced axon growth [19]. Given the role of IGF-I signaling pathways downstream from leptin (i.e., interactions between the three signaling pathways), we investigated whether underfeeding also affected leptin-induced axon growth. We therefore performed experiments similar to those described above on arcuate nucleus explants harvested from age-matched (seven-day-old) underfed pups, with evaluations of the capacity of leptin to stimulate axon growth. We found that the growth of axons from AgRP neurons from underfed pups was significantly (*p* < 0.05) stimulated by 24 h of treatment with leptin, although this effect seemed to be weaker than that in normally fed pups (see Figure 3). Leptin increased the growth of AgRP+ axons by 1.11 ± 0.02-fold relative to control conditions (Figure 4A,C): 500 ± 39 µm in control conditions and 555 ± 47 µm following leptin treatment (*n* = 5 experiments).

Total arcuate nucleus neurons were then analyzed by labeling the ubiquitous NF. Axon length was found to be 1.12 ± 0.04 times greater in leptin-treated neurons: 544 ± 42 µm in control conditions and 611 ± 48 µm following leptin treatment (*n* = 6 experiments). However, this difference was not statistically significant (Figure 4B,D), possibly due to the greater variability in total arcuate nucleus populations or the higher basal rates of axon growth observed (544 ± 42 µm in underfed controls vs. 484 45 µm in normally fed controls). In GHRH neurons, leptin treatment modulated axon length by a factor of 1.09 ± 0.05, from 394 ± 40 µm in control conditions to 428 ± 46 µm following leptin treatment (*n* = 6 experiments), although this difference was not statistically significant (Figure 4B,D). As previously observed in arcuate nucleus explants from normally fed pups, no additive or synergic stimulation of axonal growth was observed when explants from underfed pups were treated with both leptin and IGF-I (Figure 4C,D).

**Figure 5:**
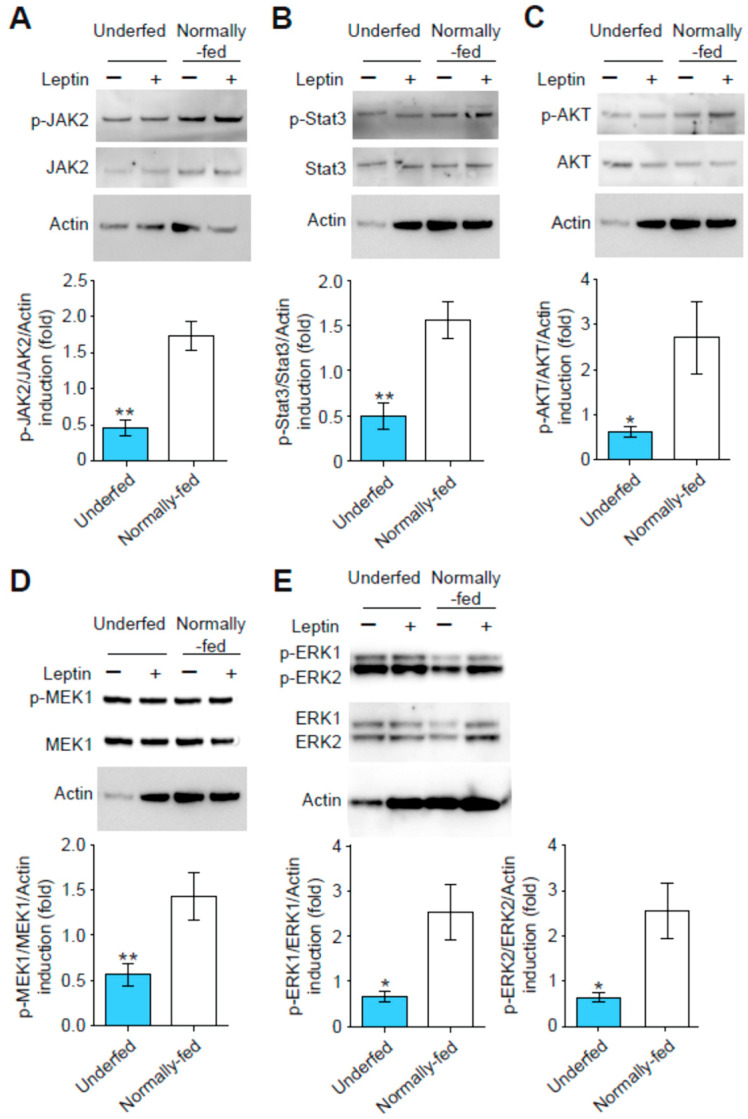
Alterations to leptin-stimulated signaling pathways in arcuate nucleus explants from underfed pups. The activation of the three signaling pathways by the exposure of seven-day explants to leptin (+) for 15 min was different in explants from underfed pups (blue bars) and in explants from normally fed pups (open bars). Data are presented as the fold induction of phosphorylated protein/total protein ratios (normalized against actin) for stimulated relative to unstimulated conditions for (**A**) phosphorylated Jak2/Jak2/actin (*n* = 5 per group), (**B**) phosphorylated Stat3/Stat3/actin (*n* = 5–7 per group), (**C**) phosphorylated-AKT/AKT/actin (*n* = 9 per group), (**D**) phosphorylated MEK1/MEK1/actin (*n* = 5–6 per group), (**E**) phosphorylated ERK1/ERK1/actin (left panel) and phosphorylated-ERK2/ERK2/actin (right panel; *n* = 8 per group). Data are presented as the mean ± SEM, with a Mann–Whitney analysis, with *: *p* < 0.05, **: *p* < 0.01.

We investigated the absence of response to leptin stimulation in GHRH neurons from underfed pups by studying the JAK-STAT, PI3K-AKT and MAPK signaling pathways. Arcuate nucleus explants from both underfed and normally fed pups were subjected to brief stimulation with leptin, and the fold-induction of phosphorylated forms (e.g., the ratio of phospho-JAK2/total-JAK2/actin) in leptin-treated explants relative to untreated explants was determined by Western blotting. The fold-induction of phosphorylated JAK2 was significantly lower in arcuate nucleus explants harvested from underfed pups than in those harvested from normally fed pups (Figure 5A).

Similar results were obtained for the induction of phosphorylated STAT3 (Figure 5B) and phosphorylated AKT in leptin-treated arcuate nucleus explants from underfed relative to normally fed pups (Figure 5C). The MAPK signaling pathway also displayed lower levels of induction for phosphorylated MEK1 (Figure 5D), phosphorylated ERK1 (Figure 5E) and phosphorylated ERK2 (Figure 5F) following leptin treatment in underfed pups relative to normally fed pups. No differences in the total levels of the proteins analyzed (i.e., total-AKT, total-Jak2, etc.) in the arcuate nucleus explants were observed between underfed and normally fed pups (data not shown). These data suggest that the JAK/STAT, PI3K/AKT and MAPK signaling pathways are all altered in arcuate nucleus explants from underfed pups.

## 4. Discussion

We show here that leptin stimulates the development of hypothalamic GHRH neurons during the first few days of life, via the JAK/STAT, PI3K/AKT and MAPK signaling pathways. In underfed pups, GHRH neurons were unable to respond to leptin stimulation despite the culture of explants in a controlled environment. This absence of response was associated with changes in the activation of the JAK/STAT, PI3K/AKT and MAPK signaling pathways. These findings support the hypothesis that linear growth may be a direct target of leptin signaling during the early postnatal period, and suggest that leptin may be a potential effector of linear growth programming by nutrition with IGF-1, despite the lack of synergy observed. Our findings also suggest that the subpopulation of GHRH neurons may present a specific response to leptin.

The involvement of leptin as a nutritional factor in hypothalamus development has been extensively studied, and it has been suggested that leptin affects the wiring of neurons, particularly those involved in the regulation of food intake, metabolism and reproduction [15,36]. Indeed, the NPY+ and POMC+ neurons of the arcuate nucleus that innervate the PVN and regulate food intake and metabolism were the first neurons shown to be sensitive to leptin signaling in the early postnatal period [15]. Leptin also has a role in reproductive function, as it has been shown to be a permissive signal for puberty, with stimulatory effects in the ventral premammillary nucleus and the arcuate NPY neurons crucial for the activation of Kiss1 neurons and puberty onset [36,37]. Leptin levels have also been associated with linear growth and differences in adult size in humans [25,26]. Consistent with this role, 45% of GHRH neurons have been reported to express the leptin receptor and to be Stat3+ following leptin stimulation [27]. However, the absence of an effect on adult size of leptin receptor knockout in mouse GHRH neurons raised questions about the potential indirect role of leptin [27]. Nevertheless, the results presented here suggest that leptin has a direct effect on axon growth in GHRH neurons, the development of which during the first week of life is crucial for the establishment of pituitary GH synthesis capacities and to program the growth trajectory. Our findings are consistent with the permanent linear growth delay associated with changes to the somatotropic axis in the Ob-R KO mouse model, in which the Ob-R is knocked out in cells expressing the GH receptor, including the GHRH neurons, the first target for the negative feedback regulation of GH secretion [28]. These findings do not rule out additional indirect growth regulation by leptin, because Ob-R deletion throughout the entire brain results in a much stronger, permanent growth delay [28].

IGF-I is one of the nutritional factors shown to modulate linear growth and to stimulate axon growth in GHRH neurons during the first week of life [19,22]. IGF-I acts through its dedicated receptor, IGF-1R, stimulating the downstream AKT/PI3K and MAPK/ERK signaling pathways. This stimulatory action appears to be specific, because insulin, which is known to improve neuronal development and to act via the same signaling pathway, has no effect on axon growth in GHRH neurons [29]. In a breast cancer model, leptin and IGF-1 have been shown to increase cell proliferation and migration, and to act in synergy in terms of the activation of their receptors, indicating interactions between these two signaling pathways [38]. Moreover, it has been suggested that leptin and IGF-1 may each cross-activate the receptor of the other (i.e., the IGF-1R and leptin-R, respectively) [38]. However, leptin and IGF-I do not seem to have synergic effects or to interact in the stimulation of axon growth in GHRH neurons, even though leptin also stimulates the PI3K/AKT and MAPK signaling pathways [23]. This may reflect a feature specific to this neuronal subpopulation. It is possible that the co-stimulation of GHRH neurons with IGF-I and leptin leads to partial desensitization and/or a negative feedback loop limiting axon growth. Indeed, IGF-1 could stimulate a negative feedback loop, notably through Stat5 and SOCS6, similar to that previously described in neural stem cells [39].

The neuronal subtype also seems to have an important effect on the action of leptin in the hypothalamus. Indeed, our findings indicate that GHRH neurons were insensitive to leptin in arcuate nucleus explants harvested from underfed pups. This finding conflicts with previous reports that the arcuate NPY and POMC neurons innervating the PVN can respond to leptin stimulation in vitro regardless of the presence or absence of leptin signaling during their development (i.e., Ob/Ob mice) [15]. However, the role of leptin in the developing hypothalamus appears to be dependent on the neuronal subpopulations and their projections. Indeed, leptin has been shown to affect the NPY glutamatergic neurons rather than the POMC neurons projecting into the autonomic area [34,40]. The specificity of neuronal subpopulations in terms of the response to leptin stimulation may depend on excitatory class [18,41] and signaling pathways. Indeed, the leptin receptor is known to activate the JAK/STAT, MAPK and PI3K/AKT signaling pathways. In particular, the MAPK/ERK signaling pathway has been shown to be crucial for the development of both NPY and POMC neurons, whereas STAT3 appears to be required for the development of POMC neurons and to have a limited effect on NPY neurons [23,34]. We found that the leptin-stimulated growth of axons in the GHRH neuron subpopulation was associated with the stimulation of the PI3K/AKT, MAPK and JAK/STAT signaling pathways. The involvement of each of these three signaling pathways was confirmed by pharmacological inhibition experiments. Moreover, these three signaling pathways also appeared to play an important role in the responsiveness of GHRH neurons to leptin linked to nutritional status, because underfeeding was associated with an abolition of leptin-induced activation of the PI3K/AKT, MAPK and JAK/STAT signaling pathways. This abolition was not associated with a change in leptin receptor gene expression in the hypothalamus of underfed pups relative to normally fed pups (data not shown). The GHRH neuron subpopulation therefore seems to display a specific response to nutritional status and nutrition-related hormones, regulating its development. A more detailed characterization of this specific subpopulation will be required to decipher the underlying mechanisms.

## 5. Conclusions

In conclusion, our results suggest a direct effect of leptin on axon growth in GHRH neurons and support the hypothesis that leptin may regulate linear growth in response to the availability of nutrients during the suckling period. They are also consistent with previous reports suggesting that leptin has a global effect on brain development, particularly in the hypothalamus, which controls all the important physiological functions of the organism [11,15,18,34].

## Figures and Tables

**Table 1:** Primary and secondary antibodies used for immunohistochemistry experiments.

Target	Name of Antibody	Manufacturer, Catalog Number	Species Raised in, Antibody Type	Dilution Used
AgRP	Agouti-Related Protein (AGRP) (82–131) Amide (Mouse) Antibody	Phoenix Pharmaceuticals, H-003-57	Rabbit, polyclonal	1/500
AgRP ^#^	Agouti-Related Protein (AGRP) (25–131) (Mouse) Antibody	R&D systems, AF634	Goat, polyclonal	1/250
GFP	Anti-GFP antibody	Abcam, ab6556	Rabbit, polyclonal	1/1000
GHRH ^#^	Anti-GHRH antibody	Proteogenix, VC-15	Rabbit, polyclonal	1/500
Leptin R	Goat anti-mouse leptin receptor	R&D system, AF497	Goat polyclonal	1/250
Neurofilament	Anti-160 kD Neurofilament Medium antibody	Abcam, ab72998	Chicken, polyclonal	1/500
Chicken IgY	Goat Anti-Chicken IgY H&L (DyLight 594)	Abcam, ab96949	Goat, polyclonal	1/400
Chicken IgY ^#^	Donkey Anti-Chicken IgY H&L (FITC)	Abcam, ab63507	Donkey, polyclonal	1/400
Goat IgG	Donkey anti-goat (DyLight 550)	Abcam, ab96932	Donkey, polyclonal	1/400
Goat IgG ^#^	Donkey anti-goat (DyLight 405)	Abcam, ab175665	Donkey, polyclonal	1/400
Rabbit IgG	Donkey Anti-Rabbit IgG H&L (DyLight 550) preadsorbed	Abcam, ab96920	Donkey, polyclonal	1/400
Rabbit IgG	Goat Anti-Rabbit IgG H&L (DyLight 488)	Abcam, ab96883	Goat, polyclonal	1/400

^#^, used for the triple IHC.

**Table 2:** Primary and secondary antibodies used for Western blot experiments.

Target	Name of Antibody	Manufacturer, Catalog Number	Species Raised in, Antibody Type	Dilution Used
Actin	anti-βactin (D6A8) HRP Conjugate	Cell Signaling, #12620	Rabbit, polyclonal	1/2000
Akt	Akt HRP Conjugate mAb	Cell Signaling, #8596	Rabbit, monoclonal	1/2000
Phospho Akt	Phospho-Akt (Ser473) (D9E) XP Rabbit mAb	Cell Signaling Technology, #4060	Rabbit, monoclonal	1/2000
Erk1/2	p44/42 MAPK (Erk1/2) (137F5) Rabbit mAb	Cell Signaling Technology, #4695	Rabbit, monoclonal	1/1000
Phospho Erk1/2	Phospho-p44/42 MAPK (Erk1/2) (Thr202/Tyr204) (D13.14.4E) XP Rabbit mAb	Cell Signaling Technology, #4370	Rabbit, monoclonal	1/2000
Jak2	Jak2(D2E12) XP	Cell Signaling 3230	Rabbit, monoclonal	1/1000
Phospho Jak2	Phospho Jak2 (Tyr 1007/1008) (C80C3) Rabbit mAb	Cell Signaling, #3776	Rabbit, monoclonal	1/1000
Mek1	Anti- Dual specificity mitogen-activated protein kinase kinase 1, MAP2K1	Boster Biological Technology, PA1376	Rabbit, polyclonal	1/3000
Phospho Mek1	Anti-Mek1 (phospho S298) antibody [EPR3338]	Abcam, ab96379	Rabbit, monoclonal	1/3000
Stat3	Stat3 (79D7)	Cell Signaling, #4904	Rabbit, monoclonal	1/2000
phospho Stat3	Phospho Stat3 (Tyr705)	Cell Signaling, #9131	Rabbit, polyclonal	1/1000
Rabbit IgG	Anti-Rabbit IgG (whole molecule)-HRP linked	Sigma-Aldrich, A0545	Goat, polyclonal	1/20,000
Rabbit IgG	Anti-Rabbit IgG (whole molecule)-HRP linked	Cell Signaling 7074	Goat, polyclonal	1/20,000

## Data Availability

The data set related to this article is available on Figshare under https://doi.org/10.6084/m9.figshare.19762123 (accessed on 12 February 2023).

## Supplementary Materials

The following supporting information can be downloaded at: https://www.mdpi.com/article/10.3390/nu15051077/s1, Table S1: Composition of the LasQCdiet Rod18-R chow.
