# Supplementary material for: Stimulation of GHRH Neuron Axon Growth by Leptin and Impact of Nutrition during Suckling in Mice
Source: Nutrients. 2023 Feb 21;15(5):1077. doi: 10.3390/nu15051077 (PMC10005278; doi:10.3390/nu15051077)
Supplement: Supplementary file 1 [file nutrients-15-01077-s001.zip › nutrients-2161637-supplementary.pdf]

Supplementary Table S1 : Composition of the LasQCdiet Rod18-R chow.

**Main nutrients**				**Amino acids per Kg**		
Protein	18.9	%		Arginine	9.0	g
Fat	5.3	%		Cysteine	4.0	g
Fibre	3.9	%		Histidine	4.5	g
Ash	7.0	%		Isoleucine	7.0	g
N-free-Extracts	53.1	%		Leucine	21.0	g
Dry matter	88.0	%		Lysine	9.0	g
				Methionine	4.5	g
Energy/kg				Phenylalanine	10.0	g
GE (gross)	16.6	MJ		Threonine	6.5	g
ME (metabolic	13.9	MJ		Tryptophan	2.0	g
				Tyrosine	7.0	g
**Minerals per kg**						
Calcium	10.0	g		**Vitamins per Kg**		
Phosphorus	6.5	g		Vit. A	15.000	I.E.
Sodium	3.0	g		Vit. D3	1.200	I.E.
Magnesium	2.5	g		Vit. E	90	mg
				Vit. K	5	mg
**Trace element per kg**				Thiamine (B1)	15	mg
Iron	200.0	mg		Riboflavin (B2)	10	mg
Iodine	4.0	mg		Pyridoxine (B6)	10	mg
Copper	15.0	mg		Cobalamin (B12)	50	mg
Cobalt	1.5	mg		Biotin	200	µg
Manganese	120.0	mg		Choline	1.000	mg
Selenium	0.2	mg		Folate	2	mg
Zinc	75.0	mg		Niacin	40	mg
				Pantothenate	20	mg
**Fatty acids per kg**						
C16 :0	7.5	g		C18 :1	13.0	g
C 18 :0	3.0	g		C18 :2	21.0	g
C20 :0	0.2	g		C18 :3	13.0	g

The LasQCdiet Rod18-R chow is provided ad libitum to mice and dedicated for gestation, lactation and growth of young animals. Ingredients are Wheat, wheat bran, corn gluten, wheat meal, corn, oats, barley, linseed oil, calcium carbonate, brewer's yeast, molasses, Vitamin-, minerals, vitamins/trace elements-mix, in descending order, respectively. It does not contain soya, fish, coating. The product is sterilized by a gamma-irradiation at minimum 21 kGy.
